# Malignant hyperthermia crisis during scoliosis surgery in a pediatric cerebral palsy patient: a case of early recognition and successful intervention

**DOI:** 10.3389/fped.2025.1601801

**Published:** 2025-06-16

**Authors:** Saleh M. Kardm, Abdulmonem Alsiddiky, Asim J. Alamri, Ravi Shankar Reddy

**Affiliations:** ^1^ Department of Surgery, College of Medicine, Najran University, Najran, Saudi Arabia; ^2^ Department of Orthopedic, College of Medicine, King Saud University, Riyadh, Saudi Arabia; ^3^ Department of Orthopedic Surgery, Prince Mohammed Bin Abdulaziz Hospital, Almadinah Almunawarah, Saudi Arabia; ^4^ Program of Physical Therapy, Department of Medical Rehabilitation Sciences, College of Applied Medical Sciences, King Khalid University, Abha, Saudi Arabia

**Keywords:** malignant hyperthermia, neuromuscular scoliosis, cerebral palsy, dantrolene, general anesthesia, pediatric scoliosis surgery

## Abstract

**Background:**

Malignant hyperthermia (MH) is a rare but life-threatening complication of general anesthesia, characterized by a hypermetabolic response that can lead to severe complications if not managed promptly. Patients with neuromuscular disorders, such as cerebral palsy and neuromuscular scoliosis, may have an increased risk of developing MH due to underlying genetic and physiological factors. Despite its rarity, early recognition and intervention are critical for patient survival.

**Case presentation:**

We present a case of MH in a 13-year-old girl with cerebral palsy and neuromuscular scoliosis undergoing elective scoliosis correction surgery under general anesthesia. The patient had no prior history of mental health (MH) susceptibility or a family history of the condition. After 90 min of surgery, she exhibited a rapid increase in end-tidal carbon dioxide (EtCO₂) to 60 mmHg, tachycardia (190 bpm), hypotension (70/40 mmHg), hyperthermia (41°C), and muscle rigidity, raising suspicion of MH. Sevoflurane inhalation was immediately discontinued, and the anesthesia circuit was changed to allow pure oxygen inhalation. The patient was treated with intravenous dantrolene, active cooling measures, forced alkaline diuresis, and correction of acid-base disturbances. These interventions successfully stabilized her vital signs, allowing the surgery to proceed safely. Postoperatively, she was transferred to the intensive care unit (ICU) for monitoring and was extubated once her condition stabilized. She was discharged on postoperative day 7 without further complications.

**Conclusion:**

This case highlights the critical need for anesthesiologists and surgical teams to remain vigilant for MH in patients with neuromuscular disorders, even in the absence of a family history. Prompt recognition and immediate intervention with dantrolene and supportive therapies are essential for a favorable outcome. Preoperative MH risk assessment, avoidance of triggering agents, and preparedness for emergency management are crucial in high-risk populations.

## 1 Introduction

Malignant hyperthermia (MH) is a rare but potentially life-threatening autosomal dominant disorder triggered by certain anesthetic agents, leading to uncontrolled hypermetabolism in skeletal muscles (1). It manifests as hyperthermia, metabolic acidosis, muscle rigidity, and cardiovascular instability (2). The overall incidence of MH in patients undergoing general anesthesia is estimated to range from 1 in 5,000 to 1 in 250,000 anesthetic exposures (3). However, in patients with neuromuscular scoliosis, studies suggest a higher incidence due to underlying genetic predispositions affecting muscle function and calcium metabolism (4).

Neuromuscular scoliosis often coexists with congenital or acquired neuromuscular disorders such as cerebral palsy, muscular dystrophies, and spinal muscular atrophy (1). These conditions are frequently associated with genetic mutations, particularly in the RYR1 gene, which encodes the ryanodine receptor—a key regulator of intracellular calcium release in muscle cells (2). Mutations in this gene are a known cause of MH susceptibility (2). Furthermore, patients with scoliosis may exhibit reduced pulmonary function and decreased chest wall compliance, leading to compromised ventilation under anesthesia, which may exacerbate metabolic disturbances associated with MH (3, 4).

Despite the known risks, there is limited literature on the real-time recognition and intraoperative management of MH in pediatric neuromuscular scoliosis patients undergoing scoliosis correction surgery. Early detection and intervention are critical in preventing fatal outcomes, yet standardized preoperative screening protocols remain inconsistent (5). This case report aims to address this gap by presenting a detailed case analysis of MH during scoliosis correction surgery in a pediatric patient with cerebral palsy and neuromuscular scoliosis. Through this case, we emphasize the importance of vigilance in high-risk populations and provide insights into the anesthetic considerations and emergency management strategies required for successful outcomes.

## 2 Case presentation

A 13-year-old girl with a history of cerebral palsy and neuromuscular scoliosis was scheduled for elective scoliosis correction surgery under general anesthesia. She had a negative family history of MH and had previously undergone multiple tendon release surgeries under general anesthesia without complications. During her prior procedures, total intravenous anesthesia (TIVA) was utilized using propofol and fentanyl without the inclusion of volatile anesthetics or succinylcholine. The absence of MH episodes during those exposures contrasts with the current case, where sevoflurane was introduced as a maintenance agent, likely serving as the triggering factor for the MH crisis. Her only significant past medical history included bronchial asthma, which was well-controlled with inhaled bronchodilators.

### 2.1 Preoperative assessment

**Figure 1:**
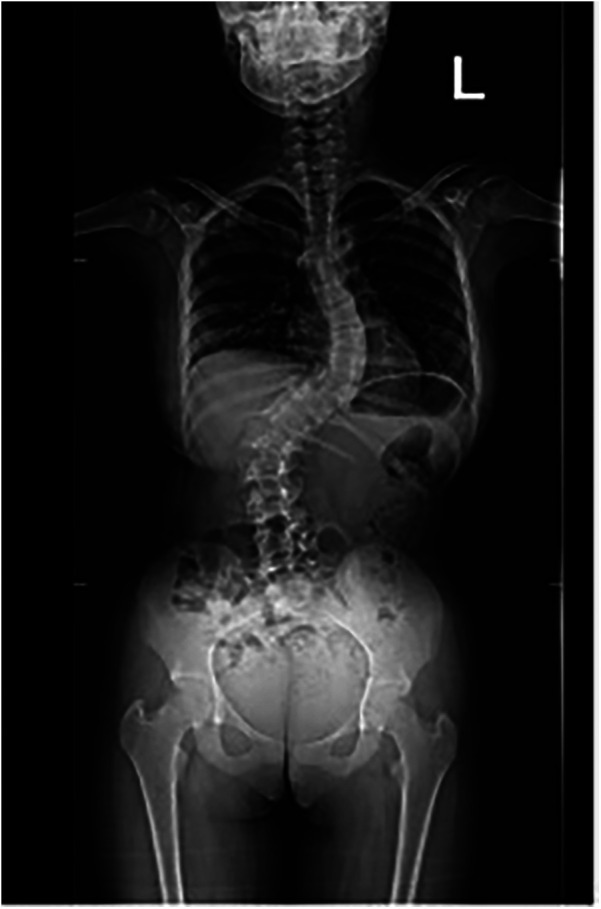
Preoperative anteroposterior radiograph showing severe neuromuscular scoliosis.

**Figure 2:**
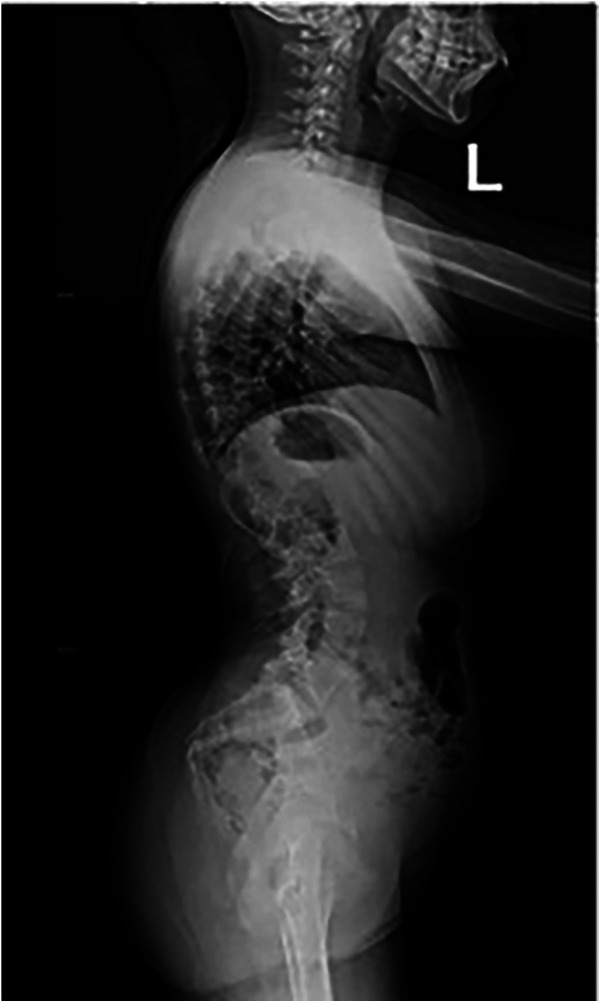
Preoperative lateral radiograph of the spine demonstrating thoracolumbar deformity and pelvic obliquity in a patient with neuromuscular scoliosis.

The patient underwent a comprehensive preoperative assessment, which included a neurological examination. The examination confirmed spasticity and hypertonia but showed no signs of progressive neuromuscular deterioration. A preoperative anteroposterior radiograph confirmed severe spinal curvature consistent with neuromuscular scoliosis (Figure 1). Lateral imaging further demonstrated thoracolumbar deformity and pelvic obliquity (Figure 2), highlighting the extent of musculoskeletal compromise preoperatively. Pulmonary function testing revealed mild restrictive lung disease, characterized by reduced forced vital capacity (FVC) but adequate baseline oxygenation, indicating a potential for intraoperative respiratory challenges. Anesthetic considerations were thoroughly discussed, focusing on anesthesia risks related to neuromuscular disorders, airway management strategies, and the avoidance of succinylcholine and volatile anesthetics whenever possible to minimize the risk of MH. Despite these considerations, MH susceptibility testing was not performed, as the patient had no known family history of MH and had previously undergone general anesthesia without complications.

### 2.2 Intraoperative course and MH episode

**Figure 3:**
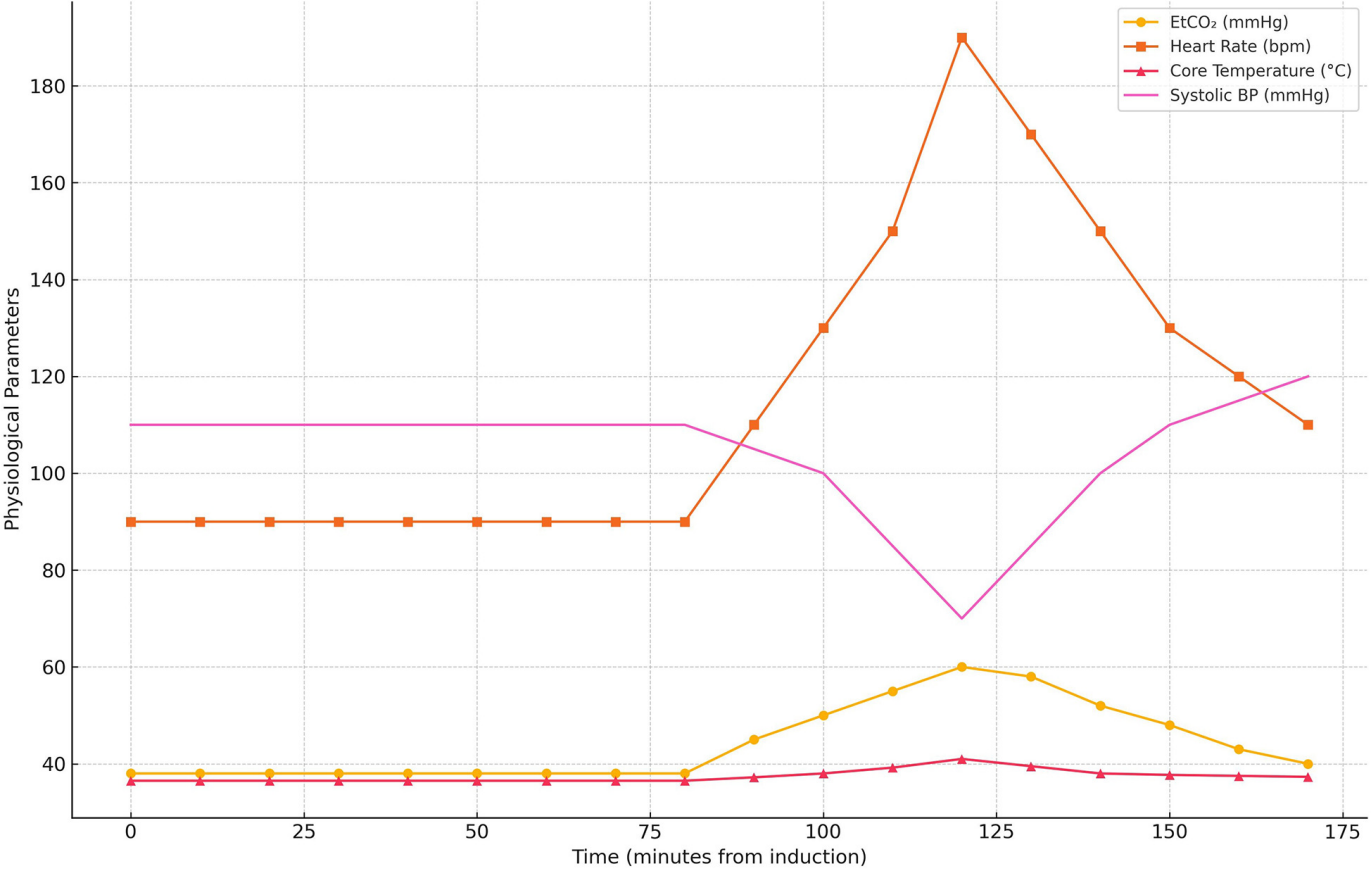
Timeline of intraoperative physiological parameters during the malignant hyperthermia crisis. The graph illustrates abrupt elevations in EtCO₂, heart rate, core temperature, and hypotension, with subsequent normalization after dantrolene administration and supportive management.

Anesthesia was induced with propofol (2 mg/kg), fentanyl (2 mcg/kg), and rocuronium (0.6 mg/kg) to facilitate endotracheal intubation. Maintenance anesthesia consisted of a propofol infusion, rocuronium, and sevoflurane. Standard intraoperative monitoring included continuous capnography, pulse oximetry, electrocardiography, invasive arterial blood pressure monitoring, and temperature measurement. Ninety minutes into the procedure, the patient exhibited a sudden increase in end- tidal carbon dioxide (EtCO₂) to 60 mmHg, despite adequate ventilation. This was followed by a rapid pulse increase to 190 bpm, hypotension (70/40 mmHg), hyperthermia (41°C), and muscle rigidity. Capnography played a crucial role in early detection, showing a rapid rise in EtCO₂, prompting immediate intervention. Based on these findings, a clinical diagnosis of MH was made. Sevoflurane was immediately discontinued, and the anesthesia machine was flushed and replaced to prevent further exposure. Anesthesia was then maintained using a propofol infusion (100–150 mcg/kg/min) with intermittent fentanyl boluses (1 mcg/kg), while neuromuscular relaxation continued with rocuronium. This non- triggering TIVA protocol was upheld for the remainder of the surgery. The crisis occurred 90 min into scoliosis correction surgery using sevoflurane, a known MH trigger, and involved deep muscular dissection, which may have contributed to the onset. The patient was pressure- ventilated with a tidal volume of 6–8 ml/kg, a respiratory rate of 14/min, and FiO₂ of 0.0.5, with a baseline EtCO₂ of 35–40 mmHg. At the onset of MH, the EtCO₂ spiked to 60 mmHg, with HR at 190 bpm, BP at 70/40 mmHg, and a core temperature of 41°C. Arterial blood gas analysis revealed a pH of 7.21, PaCO₂ of 48 mmHg, HCO_3^−^_ of 17 mmol/L, as well as elevated serum potassium (5.9 mmol/L) and lactate (5.2 mmol/L). Urine appeared dark brown within 30 min, suggesting myoglobinuria. Serum CK was measured at 9, 800 U/L, and myoglobin levels at 520 ng/ml. Circulatory support included IV crystalloids (20 ml/kg bolus), norepinephrine infusion (0.05 mcg/kg/min), and the correction of metabolic acidosis with bicarbonate. Urine pH was not measured, but the appearance was dark brown, indicating myoglobinuria. Laboratory markers of muscle breakdown were elevated, including a peak creatine kinase (CK) of 9,800 U/L and serum myoglobin of 520 ng/ml, consistent with rhabdomyolysis. Vital signs improved over 60 min: HR decreased to 120 bpm, BP increased to 120/60 mmHg, and temperature lowered to 37.7°C, enabling surgical continuation. A graphical representation of the intraoperative physiological parameters was created to depict the progression and management of the MH episode (Figure 3). The graph displays the temporal trends in EtCO₂, HR, BP, and core temperature from baseline to peak crisis and subsequent stabilization following dantrolene administration and cooling measures.

### 2.3 Management and stabilization

Sevoflurane was immediately discontinued, and the ventilator circuit, including soda lime, was replaced to allow for 100% oxygen inhalation and hyperventilation. The patient was treated with an initial intravenous bolus of dantrolene at 2.5 mg/kg, followed by two additional boluses of 1 mg/kg each, totaling 4.5 mg/kg intraoperatively. A maintenance dose of 1 mg/kg was administered every 6 h for 24 h postoperatively to prevent recurrence. Metabolic acidosis correction was achieved using a total of 60 mEq of sodium bicarbonate, administered as a 30 mEq IV bolus over 10 min, followed by a continuous infusion of 30 mEq over 1 h, guided by serial arterial blood gas measurements. The temperature decreased to 37.7°C within 30 min. Following stabilization, the surgery proceeded as planned, lasting approximately three hours without any further intraoperative complications.

### 2.4 Postoperative course and follow-up

**Figure 4:**
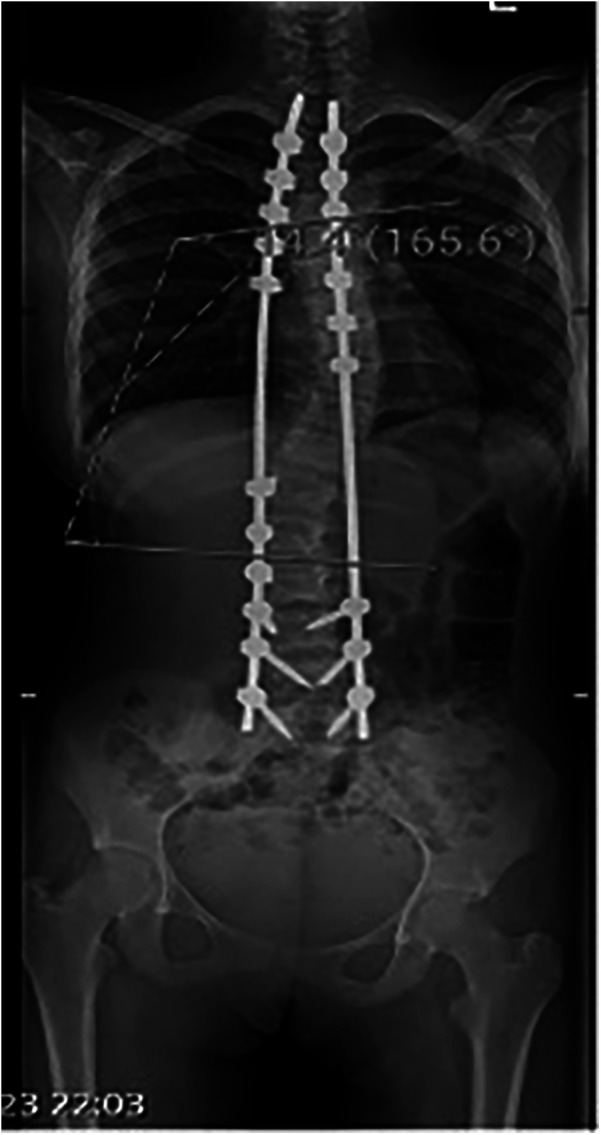
Postoperative anteroposterior radiograph demonstrating instrumented spinal fusion with satisfactory correction of coronal deformity.

**Figure 5:**
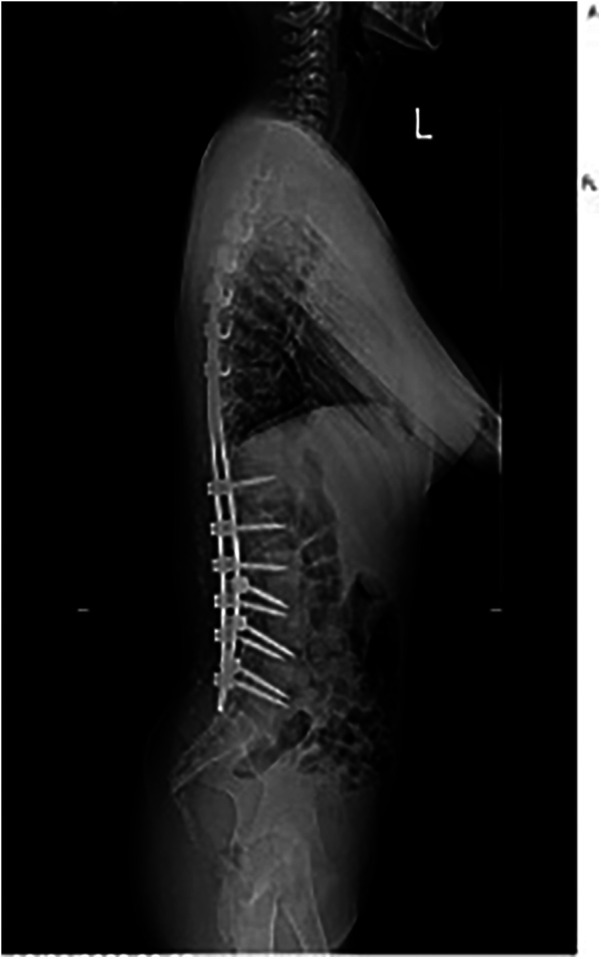
Postoperative lateral radiograph demonstrating sagittal plane alignment following posterior spinal fusion.

The patient was intubated in the intensive care unit (ICU) for continued dantrolene infusion and cooling measures. After 24 h, she was extubated once her vital signs remained stable. The dantrolene infusion was discontinued after 24 h, when the patient maintained normothermia, stable cardiovascular and respiratory parameters, and normalized metabolic markers without recurrence of symptoms, consistent with current MH treatment guidelines. No evidence of acute kidney injury or rhabdomyolysis was observed. A postoperative anteroposterior radiograph confirmed effective correction of the coronal deformity with well-aligned instrumentation (Figure 4). She was transferred to the spinal surgery ward on postoperative day 3 and discharged home on postoperative day 7 with stable vital signs. At the 2-week and 1-month follow-up, the patient demonstrated normal neurological and respiratory function with no recurrence of symptoms. The lateral postoperative view further confirmed the restoration of sagittal plane alignment (Figure 5). Genetic testing for MH susceptibility was recommended but declined. *In vitro* contracture testing (IVCT), particularly the caffeine-halothane contracture test, was also considered as a diagnostic tool but was not pursued due to its unavailability in our region and its invasive nature. Additionally, the favorable clinical response to dantrolene and the absence of recurrence reduced the urgency for further functional testing. This is consistent with current practices where genetic testing is often prioritized over IVCT when logistical constraints exist.

## 3 Discussion

In this case, the patient had no known family history of MH and had undergone a previous surgical procedure under general anesthesia without complications. However, she developed a life-threatening MH reaction during scoliosis correction surgery, demonstrating the unpredictable nature of MH susceptibility, even in patients without a prior history. The patient's clinical presentation—marked by hypercarbia, tachycardia, hyperthermia, and muscle rigidity—necessitated immediate intervention, emphasizing the need for anesthetic vigilance in neuromuscular scoliosis patients.

### 3.1 Genetic predisposition and MH risk in neuromuscular scoliosis

Mutations in the RYR1 gene, which encodes the ryanodine receptor, are a common cause of MH (5). These mutations lead to dysregulated calcium release from the sarcoplasmic reticulum, triggering uncontrolled muscle contraction and hypermetabolic responses under anesthetic exposure (6). Patients with neuromuscular scoliosis, particularly those with cerebral palsy, central core disease, or multiminicore disease, have a higher likelihood of RYR1 mutations, which predispose them to MH (7). While preoperative genetic screening is not routine, this case underscores its potential value in identifying high-risk patients, as undiagnosed MH susceptibility can lead to severe intraoperative complications (8).

### 3.2 Comparison with existing studies

Unlike the findings of Su et al. (9) and Alderson et al. (10), who reported no MH cases in their 3-year study of 400 pediatric scoliosis patients, our case demonstrates that MH can still occur in neuromuscular scoliosis patients, particularly when exposed to volatile anesthetics (9). This discrepancy underscores the importance of patient-specific risk assessments rather than relying solely on general epidemiological trends. Additionally, studies such as Rosenberg et al. (1) found that 17% of neuromuscular scoliosis patients tested positive for MH susceptibility, compared to only 1% of non-scoliotic patients, reinforcing the higher risk in this patient population (1, 11). Research by Rendu et al. (12) and Riazi et al. (13) suggest that abnormal calcium regulation in skeletal muscle cells may explain the increased vulnerability to MH in patients with neuromuscular disorders, further supporting the need for enhanced perioperative monitoring in scoliosis surgeries (13). This case aligns with previous reports where RYR1 mutations were implicated in patients with neuromuscular conditions who developed MH despite no prior known susceptibility (13). Although neither genetic testing nor in IVCT was performed, the diagnosis of MH in this case was made based on clinical criteria. The patient exhibited hallmark signs of malignant hyperthermia (MH), including a sudden rise in end-tidal CO₂ (60 mmHg), hyperthermia (41°C), generalized muscle rigidity, severe metabolic acidosis, elevated creatine kinase (CK) levels, and myoglobinuria, all of which occurred intraoperatively after exposure to a known trigger (sevoflurane). Using the Clinical Grading Scale (CGS) proposed by Larach et al. (14), the calculated score exceeded the threshold for a rank of “almost certain,” strengthening diagnostic confidence. Differential diagnoses such as muscular dystrophy, neuroleptic malignant syndrome, and isolated rhabdomyolysis were considered but ruled out based on clinical context, medication history, and the specific timing and constellation of symptoms. The rapid response to dantrolene further supported MH as the most plausible diagnosis.

### 3.3 Clinical recommendations

To enhance patient safety and minimize the risk of MH in neuromuscular scoliosis patients undergoing general anesthesia, we propose several key recommendations. Preoperative MH screening should be considered for all patients with neuromuscular scoliosis, particularly those undergoing major surgical procedures under general anesthesia, to assess potential susceptibility and mitigate intraoperative risks. When selecting anesthetic agents, non-triggering options such as propofol and dexmedetomidine should be prioritized over volatile anesthetics and succinylcholine, both of which are known MH triggers and can significantly increase the likelihood of a hypermetabolic crisis. Additionally, anesthetic teams must have dantrolene readily available at all times and be trained in rapid MH response protocols, ensuring immediate and effective intervention in the event of a suspected reaction. Proper preparation, awareness, and protocol adherence are critical in improving patient outcomes and preventing life-threatening complications in high-risk populations.

Preoperative risk assessment should include a detailed family and anesthetic history, paying special attention to previous unexplained anesthetic events or known neuromuscular diagnoses. High-risk patients include those with congenital myopathies (central core disease), confirmed or suspected RYR1 mutations, or prior intraoperative complications suggestive of MH. Genetic testing should be pursued when available. Although caffeine-halothane contracture testing is considered definitive, it remains largely inaccessible in many countries, including Saudi Arabia, where clinical evaluation and genetic screening are used to guide risk stratification.

In response to this case, our institution has revised anesthetic protocols for patients with neuromuscular scoliosis and cerebral palsy. Inhaled anesthetics and succinylcholine are now avoided, and total intravenous anesthesia using agents such as propofol and dexmedetomidine is the standard of care. This policy change reflects evolving best practices and aligns with literature supporting non-triggering anesthetic strategies in MH-susceptible populations. The diagnostic and therapeutic approach adopted in this case is consistent with current expert guidelines (15). The European Malignant Hyperthermia Group (EMHG, 2020) and the Malignant Hyperthermia Association of the United States (MHAUS, 2023) recommend prompt recognition based on clinical signs, discontinuation of triggering agents, administration of dantrolene, active cooling, and supportive care as the first-line treatment (1). Our reliance on the Clinical Grading Scale to support diagnosis aligns with its validated use in malignant hyperthermia assessment, particularly when confirmatory genetic or contracture testing is unavailable. These recommendations are also reflected in recent literature reviews and international expert consensus Cong et al. (16), which emphasize vigilance in high-risk populations, preparedness with dantrolene, and the avoidance of volatile anesthetics and depolarizing neuromuscular blockers in susceptible individuals.

## 4 Conclusion

This case report emphasizes the critical importance of vigilance for MH during scoliosis correction surgery, particularly in patients with cerebral palsy and other neuromuscular disorders. Prompt recognition and immediate intervention with dantrolene, cooling measures, and metabolic stabilization are essential for a favorable outcome. Larger cohort studies are required to establish risk factors for MH in neuromuscular scoliosis patients and to evaluate the role of genetic screening as a routine preoperative measure. Given the unpredictable nature of MH, preoperative risk assessment, avoidance of triggering agents, and preparedness for emergency management should be standard practice in high-risk populations. Anesthesiologists and surgical teams must ensure dantrolene availability, early detection via intraoperative monitoring, and strict adherence to MH crisis protocols. Further research is needed to better understand the underlying mechanisms of MH, identify high-risk patient profiles, and develop standardized screening and prevention strategies to improve surgical safety in this vulnerable population.

## Data Availability

The original contributions presented in the study are included in the article/Supplementary Material, further inquiries can be directed to the corresponding author.
